# Practitioners' Bookshelf

**Published:** 1891-10-17

**Authors:** 


### PRACTITIONERS' BOOKSHELF.

### HYDROPHOBIA AND PASTEUR.*

It has been very difficult indeed for busy practitioners to form any definite conclusions as to the value of Pasteur's inoculations for hydrophobia. Of the value of Pasteur's work, as a whole, no truly scientific man has a doubt; but it is this great position of Pasteur which makes it so difficult to disbelieve in anything which is advocated in his great name. But if there is much to be said in support of M. Pasteur's treatment of "La Rage," there is a great deal to be said on the other side, and this adverse criticism is very ably put forth and supported by Dr. Lutaud. In examining the method he considers the following six questions: 1. Is the dog which bites the patient mad? 2. Is the patient treated actually attacked with hydrophobia? 3. How is it that the number attacked has been increased a hundredfold in three months? 4. What is wolf madness? 5. Why is the madness more severe when the bite is deep? 6. How is it that the rabid virus does not give rise to any reaction, local or general? Before advising patients to undergo the treatment, this treatise of Dr. Lutaud may be consulted with advantage; it may save the patient a journey and something worse.

### THE NATURE AND TREATMENT OF DIPHTHERIA.†

This, the third edition of a work now well known, has little need of recommendation. The pathology is up to date and the treatment correspondingly abreast of the times. It is the work of a practical man of large experience, and is well worthy of perusal by the busy practitioner. These are the books we want.

### THE TREATMENT OF TYPHOID FEVER.‡

This little brochure is the subject of a lecture delivered in King's College Hospital, and published in the *Lancet*, together with additional observations. We have read Dr. Burney Yeo's small treatise with much pleasure, and believe that it gives the most complete and succinct account of the present state of the antiseptic treatment of typhoid fever that has yet been published.

* "Etudes sur La Rage," Dr. Lutaud, Rédacteur en Chef de *Journ. de Médecine de Paris*. Second ed., pp. 440. Paris: *Journal de Médecine*, 1891.

† "The Nature and Treatment of Diphtheria," by R. W. Parker, Senior Surgeon to the East London Hospital for Children, Pp. 182. Illustrated. London: H. K. Lewis, 1891.

‡ "The Treatment of Typhoid Fever, especially by Antiseptic Remedies." J. Burney Yeo, M.D., F.R.C.P.; pp. 70. London: Cassell and Co., 1891.

---

Those who are curious as to the origin of the outbreak of influenza during the two past years and as to its vagaries will find some interesting information in the "Transactions of the Second Session of the Intercolonial Medical Congress of Australasia," published by Stillwell and Co., of Melbourne. The epidemic of influenza which visited Australasia in 1885 was known there as "fog fever." It appeared in May, and reached its height for the year in August, recurring each subsequent year with increased virulence in the winter months. This is far from encouraging news to us here. We are not aware of the reason of influenza being called "fog fever," but it is remarkable that the past winter was notorious for fogs, not in London alone, but throughout the country. A repetition of the joys of last winter in London will solve beyond any doubt the problem as to whether life is worth living.

